# A Prognostic Gene Signature for Hepatocellular Carcinoma

**DOI:** 10.3389/fonc.2022.841530

**Published:** 2022-04-27

**Authors:** Rong Chen, Meng Zhao, Yanli An, Dongfang Liu, Qiusha Tang, Gaojun Teng

**Affiliations:** ^1^ Department of Oncology, Zhongda Hospital, Nanjing, China; ^2^ School of Basic Medicine, Zhengzhou University, Zhengzhou, China; ^3^ Medical School of Southeast University, Nanjing, China; ^4^ Department of Radiology, Medical School of Southeast University, Nanjing, China

**Keywords:** hepatocellular carcinoma, bioinformatics analysis, immune-related prognostic genes, immune cells, gene signature

## Abstract

Hepatocellular carcinoma is the third most common cause of cancer-related deaths in China and immune-based therapy can improve patient outcomes. In this study, we investigated the relationship between immunity-associated genes and hepatocellular carcinoma from the prognostic perspective. The data downloaded from The Cancer Genome Atlas Liver Hepatocellular Carcinoma (TCGA-LIHC) and the Gene Expression Omnibus (GEO) was screened for gene mutation frequency using the maftools package. Immunity-associated eight-gene signature with strong prognostic ability was constructed and proved as an independent predictor of the patient outcome in LIHC. Seven genes in the immune-related eight-gene signature were strongly associated with the infiltration of M0 macrophages, resting mast cells, and regulatory T cells. Our research may provide clinicians with a quantitative method to predict the prognosis of patients with liver cancer, which can assist in the selection of the optimal treatment plan.

## Introduction

Hepatocellular carcinoma (HCC) is the fifth most common cancer in the world (1, 2). China accounts for 55% of new HCC cases and HCC-related deaths annually (3). Standard treatment approaches for HCC include surgery, liver transplantation, targeted therapy, radiotherapy, immunotherapy, and chemotherapy; however, the therapeutic effect is still not satisfactory (4). Thus, in China a 5-year survival rate for patients with HCC is only 14.1% (5). The overall poor outcome can be attributed to the fact that patients are already at the advanced stage when diagnosed and only less than 30% of them can be operated (6). Therefore, in order to improve patients’ survival, it is important to explore new diagnostic and therapeutic targets, including disease-specific biomarkers and prognostic molecular models.

Mutations in the TP53 gene encoding an important tumour suppressor protein are commonly found in diverse human cancers (7–9). Wild-type TP53 can activate apoptosis-related pathways to induce cancer cell death and prevent tumour growth (10), whereas loss-of-function mutations in the TP53 gene can induce uncontrolled tumour cell proliferation (11, 12), as shown for oesophageal cancer (13, 14). In breast cancer, the frequency of TP53 mutations is as high as 80%, which exceeds even that of BRCA1 mutations (15) and which can account for shorter lifespan of patients with mutated TP53 (16). In high-grade ovarian cancer, the TP53 mutation rate is approximately 97% (17). Adavosertib can increase the chemotherapeutic drug sensitivity of cancer cells harbouring TP53 mutations (18, 19), and it was reported that in patients with platinum-sensitive ovarian cancer, adavosertib combined with paclitaxel and carboplatin can improve progression-free survival (PFS).

TP53 mutations are also very common in HCC and have been detected in 13–48% of patients (20–24). Patients with HCC and mutated TP53 had shorter overall and relapse-free survival (25). Previous studies indicate that the TP53 mutation status is associated with distinct immune reactions (26, 27); thus, in HCC TP53 genetic alterations resulted in decreased immune response (28). It has been reported that mutations in both low-density lipoprotein receptor-related protein 1B (LRP1B) and TP53 may be a prognostic biomarker predicting a better effect of immunotherapy in patients with HCC (29). The predictive value of the TP53 mutation status was also shown in the treatment and prognosis of other cancers. Thus, in squamous cell carcinoma of the head and neck (HNSCC), mutations in methylguanine-DNA methyltransferase and TP53 were related to a poorer prognosis (30). Recent studies indicate that the cooperative effect of poly (ADP-ribose) polymerase (PARP) inhibitors and ionic radiation or chemotherapy depends on the TP53 function (31, 32). Moreover, reactivation of mutant TP53 combined with olaparib resulted in more efficient inhibition of tumour growth in the preclinical model of triple negative breast cancer with a TP53 mutation (33). PARP inhibitors showed activity in a subset of colorectal cancer cell lines and preservation of the TP53 function may increase the likelihood of a favourable response (34).

As TP53 mutations play a significant role in many types of cancers including HCC, previous studies have been limited to the investigation of this particular gene (22, 23, 28). However, it is established that cancer is a heterogeneous multi-stage disease caused by the interaction of numerous gene products and signalling factors. Therefore, an integrative functional genomics approach should help in deciphering the molecular features of liver cancer. In previous studies, either the sample size was insufficient, which undermined the reliability of conclusions (35), or the records on baseline clinical features and therapeutic regimens and even the information included in the dataset were incomplete (36). Therefore, comprehensive testing and analysis are required to identify more reliable diagnostic biomarkers and therapeutic targets in HCC.

In this study, we used The Cancer Genome Atlas (TCGA) and Gene Expression Omnibus (GEO) databases to obtain and screen highly mutated genes in primary liver cancer, construct an immune-related gene signature, and explore the relationship between immune cells and patient prognosis.

## Materials and Methods

### Acquisition of Liver Hepatocellular Carcinoma (LIHC) Data and Screening of Highly Mutated Genes

The gene expression data on 364 cases of LIHC were downloaded from TCGA database (37) using RTCGAToolbox (38) and used as a training set. The LIHC gene chip and clinical survival data of 115 LIHC cases contained in the GSE76427 dataset (39) were downloaded from the GEO database (40) and used as the validation set. Maftools (41) was used to identify the top 20 highly mutated genes and visualize mutations and their frequencies in all samples, which were then divided into two groups according to the presence of mutations in the gene with the highest mutation frequency.

### Gene Set Enrichment Analysis (GSEA) and Gene Set Variation Analysis (GSVA)

To determine the pathways differentially expressed between patient groups, we performed GSEA, a computational method to detect functionally relevant genes (42, 43) and GSVA, a non-parametric approach to calculate sample-wise gene set enrichment scores for gene expression data (44). For enrichment analysis, we used GSEA 4.0.3 software and file ‘c2.all.v.7.1.symbols.gmt’ as the reference gene set, and performed 1,000 genome replacements to determine the standardized enrichment score for each analysis; P-values and false discovery rates less than 0.05 were considered to indicate statistical significance. File ‘h.all.v7.1.symbols.gmt’ was used as the reference gene set for GSVA performed with clusterProfiler (45). P-value less than 0.05 indicated statistical significance.

### Determination of the Immunity-Associated Gene Signature

Univariate Cox regression was used to analyze the association between immunity-related genes and the prognosis of patients with LIHC; forest plots were constructed for visualization. Screening of immune genes correlated with disease prognosis was used as the basis for signature construction; P <0.05 was the selection criterion. We applied machine learning methods and Lasso regression, which is widely used in search of prognostic biomarkers (46), to generate a new gene combination for each iteration; 1,000 Lasso regressions were performed on candidate genes and the best gene signature was determined based on the area under the curve (AUC). Next, we calculated the risk score for each patient according to the gene expression level and divided patients into groups. The optimal prognostic immune-gene signature was verified by Cox regression analysis.

### Gene Ontology (GO) and Kyoto Encyclopaedia of Genes and Genomes (KEGG) Analyses

GO is widely used to annotate gene functions (47), and KEGG is a common method to analyze pathway enrichment (48). For studying the functions of genes associated with LIHC prognosis and the related molecular mechanisms, an R package clusterProfiler (45) was applied to perform GO and KEGG analyses.

### External Validation of the Immunity-Related Gene Signature

The risk score for each sample in the validation set was calculated according to the optimal gene signature and used to assign patients to high- and low-risk groups. The receiver operating characteristic (ROC) curve at different time points was used to analyze the prognostic potential of the optimal gene signature in the validation set.

### Clinical Subgroup Analysis and Nomogram Construction

We grouped patients with LIHC according to clinical characteristics (gender, age, pathological stage, TNM stage, and metastasis) and performed Kaplan-Meier analysis on the samples in each group. Next, we constructed a nomogram including the predictive information on clinical features and gene signature.

### Analysis of Correlation Between Immune Cell Infiltration and the Optimal Gene Signature

An online CIBERSORT tool (49) was used to analyze the distribution and infiltration of 22 types of immune cells in the high- and low-risk groups. Principal Component Analysis (PCA) was applied to the data to determine the difference in immune cell infiltration between the two groups. We also evaluated the inter-group differences in the composition, interaction, and infiltration of the 22 immune cell types. Further, the association between immune cell infiltration and LIHC prognosis was explored using Kaplan-Meier analysis.

### q-RCR

Total RNA from cells was extracted with TRIzol reagent (Thermo Fisher Scientific, 15596026) following the manufacturer’s instructions. Complementary DNA (cDNA) was synthesized and PCRs with cDNA as template were performed using a real-time detector (Analytik Jena AG, qTower 3.2G; Jena, Germany) using BeyoFast SYBR Green qPCR Mix (Bio-Rad, 1708882AP, Shanghai, China). The primer sequences were as follows: GAPDH Forward: 5’-ACAGCCTCAAGATCATCAGC-3’; GAPDH Reverse: 5’-GGTCATGAGTCCTTCCACGAT-3’; CCR3 Forward: 5’- CACAAGCCAGGGAGAAGTGA-3’; CCR3 Reverse: 5’- TTTTCACAGAGCAGGCCCAC -3’; CHGA Forward: 5’- CAGCGGTTTTGAAGATGAACTC -3’; CHGA Reverse: 5’- ACTTTTCTCTGCCTCCTTGGAA -3’; EPO Forward: 5’- GCTGCATGTGGATAAAGCCG -3’; EPO Reverse: 5’- TGATTGTTCGGAGTGGAGCA -3’; LECT2 Forward: 5’- CTGCTCAAAGAAGTCAGAGGC -3’; LECT2 Reverse: 5’- GCGTACACAGTAGATCCAGCA -3’; NROB1 Forward: 5’- AGGGGGTAAAGAGGCGCTA -3’; NROB1 Reverse: 5’- CTTGATTTGTGCTCGTGGGC -3’; S100A9 Forward: 5’- GGAACGCAACATAGAGACCA -3’; S100A9 Reverse: 5’- GATCTTTTCGCACCAGCTCTT -3’; SEMA4F Forward: 5’- CCTGCCTCCCACACACTTTA -3’; SEMA4F Reverse: 5’- ACCATCCAGTCAATCCTGCG -3’; SPP1 Forward: 5’- CAAATACCCAGATGCTGTGGC -3’; SPP1 Reverse: 5’- TGGTCATGGCTTTCGTTGGA -3’. Transcript levels were normalized against GAPDH levels as an internal reference and were evaluated using the 2-ΔΔCt method. All experiments were repeated three times.

### Statistical Analysis

All statistical analyses were performed in R package. Cox regression analysis was applied to verify the association of patient survival with the gene signature and the expression of each signature gene. Kaplan-Meier analysis was used to evaluate the survival of patients in the high- and low-risk groups. Pearson correlation analysis was performed to determine the correlation between the prognostic gene signature and infiltration of prognosis-related immune cells. P-values and false discovery rates less than 0.05 were considered to indicate statistical significance.

## Results

### Mutant Genes in LIHC

**Figure 1:**
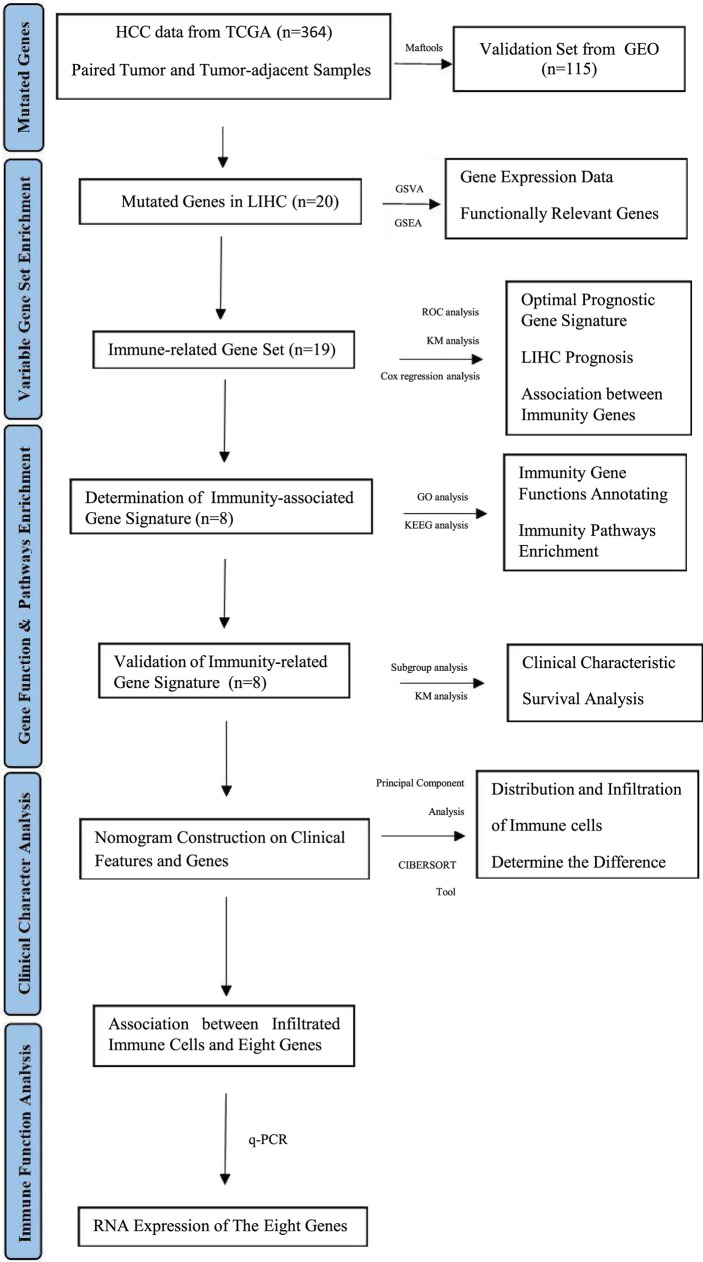
Bioinformatics Algorithm of prognostic gene set for hepatocellular carcinoma.

**Figure 2:**
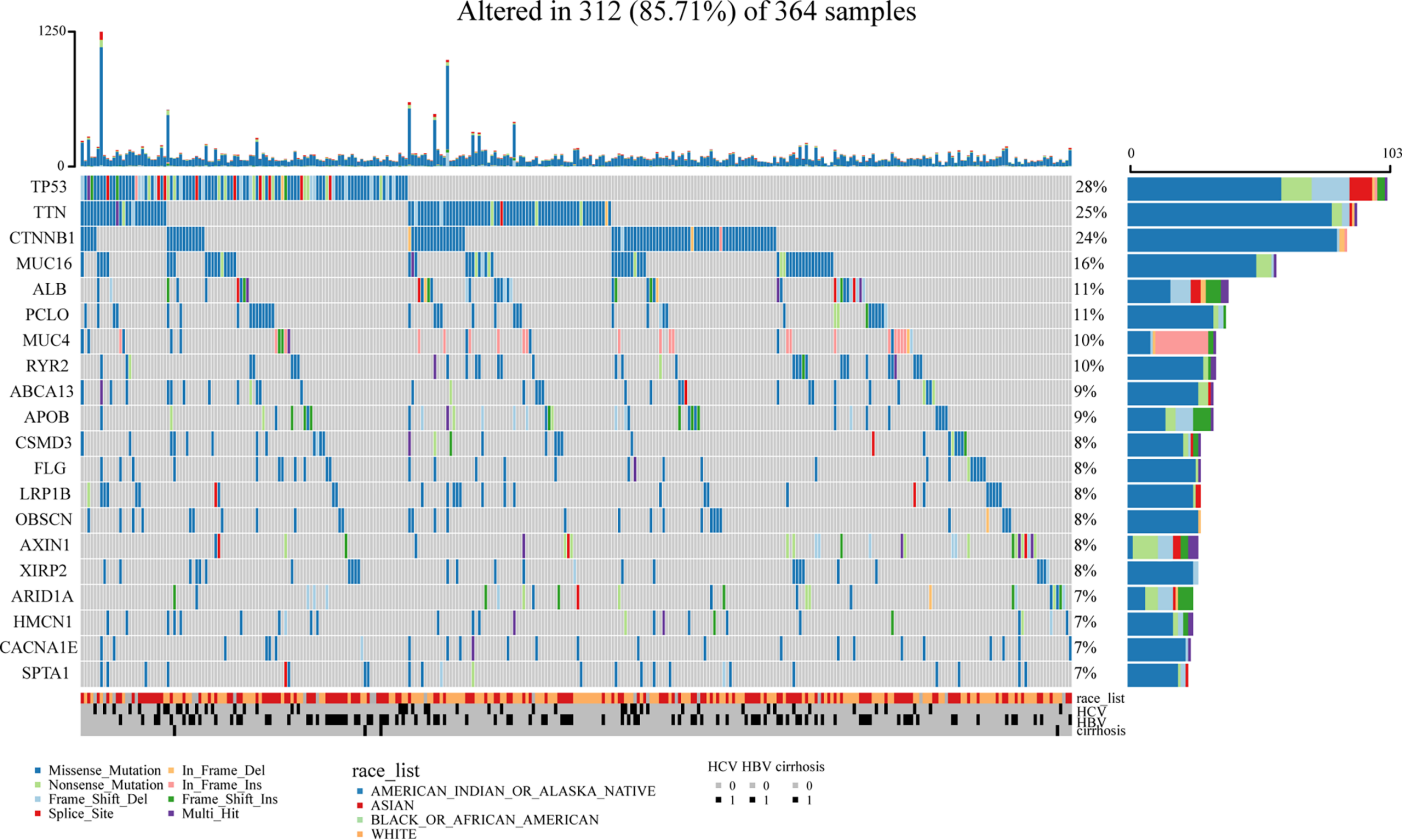
Identification of mutated genes. The maftools package was used and we identified the top 20 highly mutated genes in LIHC. TP53 had the highest mutation frequency among them.

A flowchart of this study is shown in **Figure 1**. Using the maftools package (41), we identified the top 20 highly mutated genes in LIHC: SPTA1, CACNA1E, HMCN1, ARID1A, XIRP2, AXIN1, OBSCN, LRP1B, FLG, CSMD3, APOB, BCA13, RYR2, MUC4, PCLO, ALB, MUC16, CTNNB1, TTN, and TP53; among them TP53 had the highest mutation frequency (**Figure 2**).

### GSEA and GSVA Results

According to the TP53 mutation status, a total of 364 samples were divided into the TP53 and NO_TP53 groups. GSEA showed that four immune-related pathways: Hoffmann-large-to-small-pre-bil-lymphocyte-up, croonquist-IL6-deprivation-dn, mori-large-pre-bil-lymphocyte-up and lee-early-t-lymphocyte-up were enriched in the TP53 group (**Supplementary Figure 1A**). GSVA confirmed that many immune-related KEGG pathways including myc-targets-v1, orc1-signalling, ical-junction, folded-protein-response, apoptotic-spindle, f-targets, 3-pathway, m-checkpoint, response-up, c-targets-v2, glycolysis, apoptosis, 2-stat5-signalling, 3k-akt-mtor-signalling, and complement were activated were enriched in the TP53 group, which further indicating that the activation of TP53 might participate in the process of immune process (**Supplementary Figure 1B**).

### Identification of Immune-Related Prognostic Genes and Signature Construction

**Figure 3:**
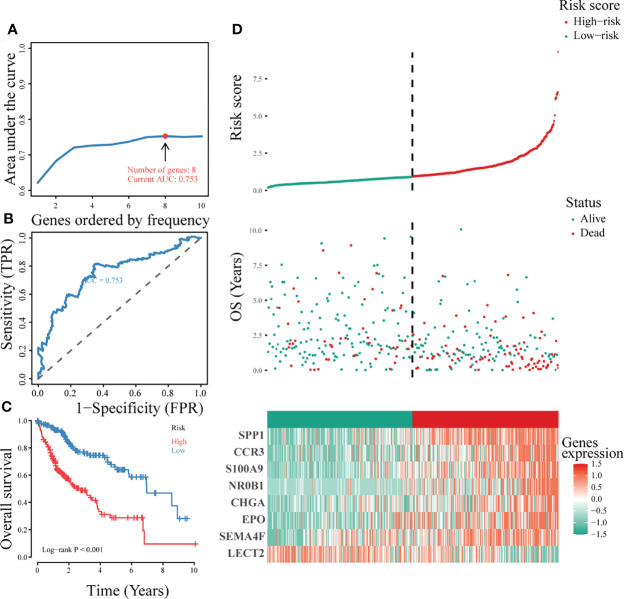
Construction of the optimal immune gene signature associated with LIHC prognosis. **(A)** Iterative Lasso Cox regression analysis used to construct the immune gene signature based on the size of the AUC. **(B)** ROC analysis of the optimal immunity-associated gene signature. **(C)** Kaplan-Meier curves of different risk groups. **(D)** The risk factor association diagram showing risk score distribution, survival status, and expression of the signature genes in the two risk groups.

A single-factor Cox regression model showed that 19 immunity-related genes: BIRC5, CALCR, CCR3, CHGA, COLEC12, CXCL8, EPO, FABP6, FGF9, IKBKE, MAPT, NR0B1, S100A11, S100A2, S100A9, SEMA4F, SPP1, STC2, and TNFRSF11B were associated with LIHC prognosis (**Supplementary Figure 2**). For the accuracy of predicting the optimal gene signature for LIHC, we performed iterative Lasso Cox regression analysis, which identified a prognostic signature comprising eight genes: LECT2, SEMA4F, EPO, CHGA, NR0B1, S100A9, CCR3, and SPP1 (**Figure 3A**). ROC analysis showed that the eight-gene signature had a good predictive ability (**Figure 3B**), whereas Kaplan-Meier analysis revealed that the overall survival of patients in the low-risk group was significantly better than that in the high-risk group (P < 0.001; **Figure 3C**). **Figure 3D** shows the survival status, risk score distribution, and expression of the signature genes. The mortality rate was significantly higher in the high-risk than in the low-risk group and each signature gene was differentially expressed in the two groups. Cumulatively, these results indicated that the signature comprising eight immunity-associated genes could be a significant prognostic indicator in LIHC.

### Verification of the Optimal Immune-Associated Gene Signature in the External Validation Set

Analysis of the survival status, risk score distribution, and gene expression of eight-gene signature in the validation set (**Supplementary Figure 3A**) confirmed that the prognosis of patients in the low-risk group was significantly better than that in the high-risk group, thus verifying the prognostic ability of the signature. Kaplan-Meier survival analysis showed that the eight-gene signature could predict the prognosis for patients with LIHC in the external verification set (P = 0.0017) (**Supplementary Figure 3B**). ROC analysis of survival prognosis indicated that the eight-gene signature had a strong ability to predict 3-year (AUC = 0.71), 5-year (AUC = 0.78), and 7-year (AUC = 0.68) survival of patients with LIHC (**Supplementary Figure 3C**). Comparison of the immune-related eight-gene signature with the established LIHC prognostic biomarkers showed that the prediction based on the gene signature was significantly more reliable (**Supplementary Figure 3D**).

### Independent Predicting Ability of the Eight-Gene and Construction of a Prognostic Nomogram

**Figure 4:**
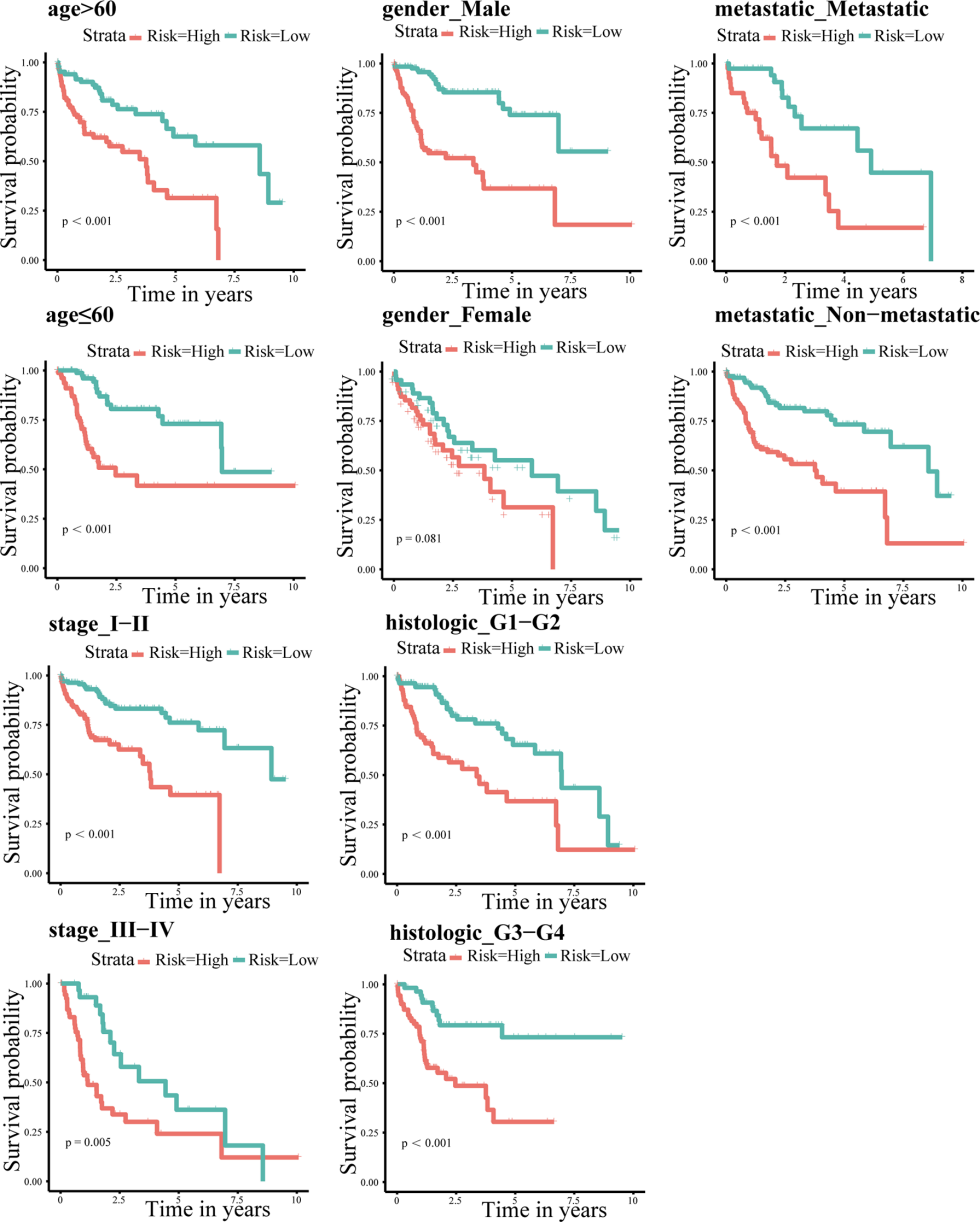
Kaplan-Meier survival analysis according to individual clinicopathological characteristics (age, gender, metastasis, and TNM and pathological staging). Red and green indicate high- and low-risk groups, respectively.

**Figure 5:**
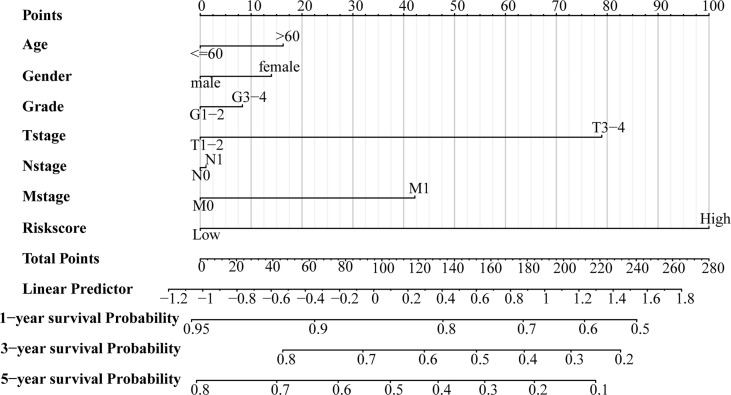
A prognostic nomogram for the overall survival of patients with LIHC.

Patients were regrouped and Kaplan-Meier survival analysis was performed based on clinicopathological characteristic. The results indicated that even if the clinical features were regrouped, the survival in the high-risk group was always poor (P < 0.05 for all; **Figure 4**). In addition, we combined clinicopathological characteristics and the immune-related eight-gene signature and constructed a prognostic nomogram (**Figure 5**), which could aid in the clinical decision regarding the treatment plan.

### Immune Cell Infiltration in the Two Risk Groups

PCA revealed the difference in immune cell infiltration between the two risk groups (**Supplementary Figure 4A**). The results of correlation analysis showed that the infiltration of CD8+ T cells was positively correlated with that of regulatory T cells (Tregs), M1 macrophages, and follicular helper T cells (**Supplementary Figure 4B**). However, the infiltration of naïve B cells was negatively correlated with that of CD8+ T cells, macrophages (M0, M1, and M2), monocytes, resting dendritic and NK cells. Immune cell interaction network revealed that M0 macrophages, activated NK cells, naïve B cells, and resting CD4+ memory T cells had the strongest, whereas activated dendritic and mast cells, naïve CD4+ T cells, and resting dendritic cells – the weakest association with other immune cells (**Supplementary Figure 4C**). Immune cell composition analysis revealed that activated NK cells had the highest infiltration rate and activated dendritic cells – the lowest infiltration rate (**Supplementary Figure 4D**).

### Association of Immune Cell Infiltration With LIHC Prognosis and the Eight-Gene Signature

**Figure 6:**
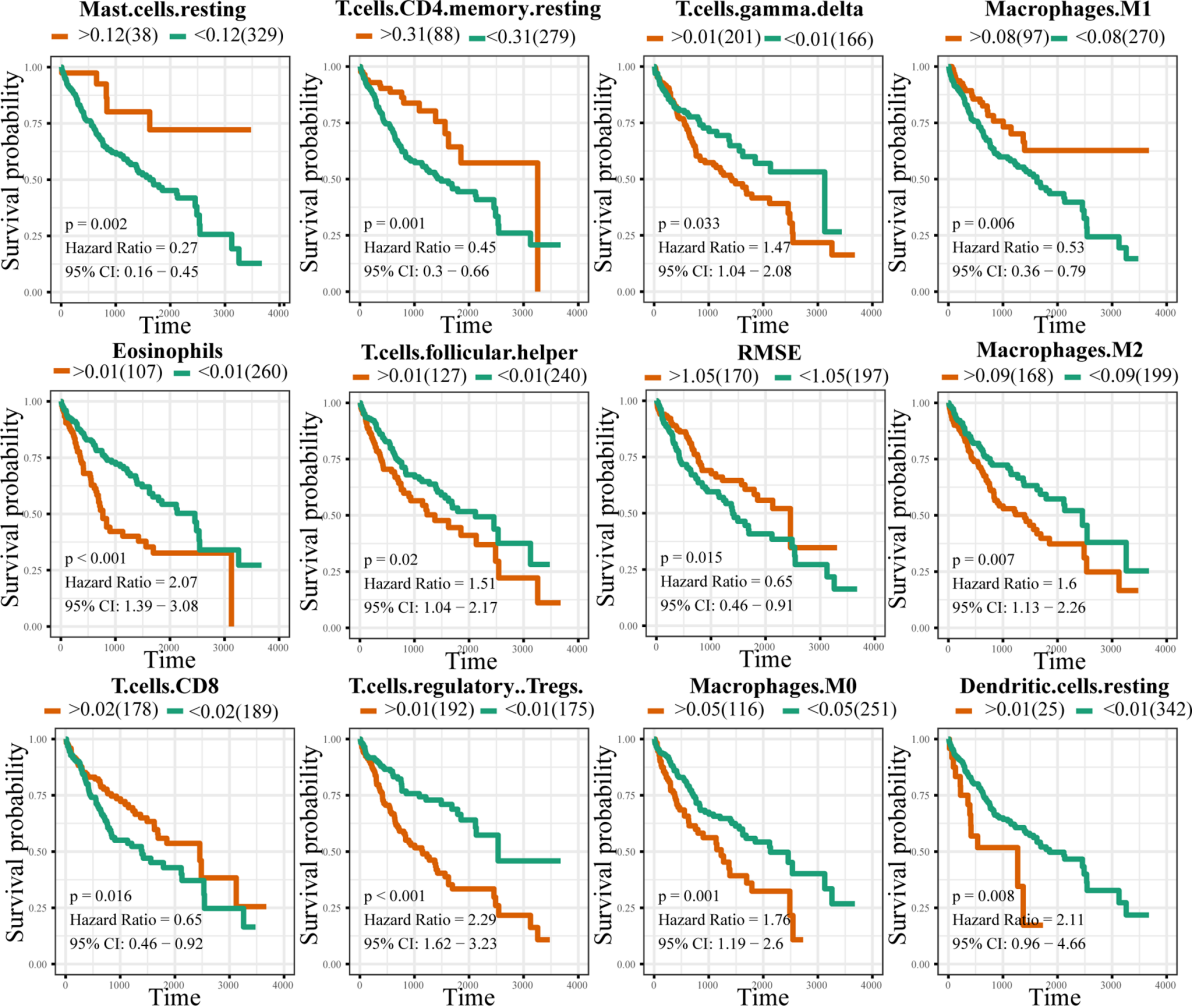
Correlation between immune cell infiltration and LIHC prognosis. Orange and green colours indicate the high- and low-risk groups, respectively.

**Figure 7:**
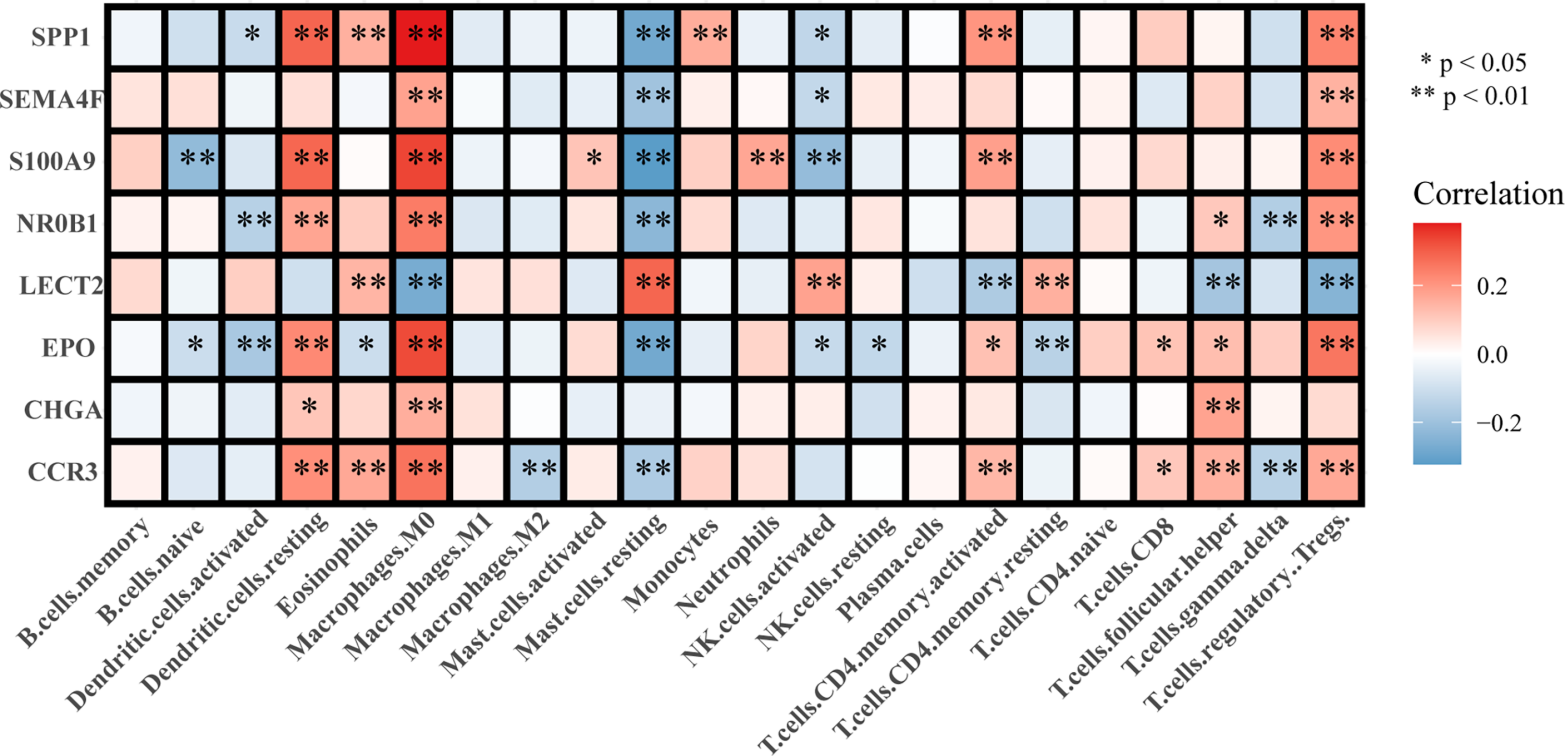
Correlation between the signature genes and immune cell infiltration.

Analysis of the correlation between immune cell infiltration and prognosis showed that the infiltration of gamma delta T cells, eosinophils, and M0 and M2 macrophages indicated a poorer prognosis, whereas that of CD8+ T cells, M1 macrophages, and NK cells suggested a better prognosis for patients with LIHC (**Figure 6**). The results of the constructed correlation heat map for the signature genes revealed that M0 macrophages, resting mast cells, and Tregs showed negative correlation with CCR3, EPO, NR0B1, S100A9, SEMA4F, and SPP1, and positive correlation with LECT2 (**Figure 7**).

### The Relative RNA Expression Level of LECT2, SEMA4F, EPO, CHGA, NR0B1, S100A9, CCR3, and SPP1

**Figure 8:**
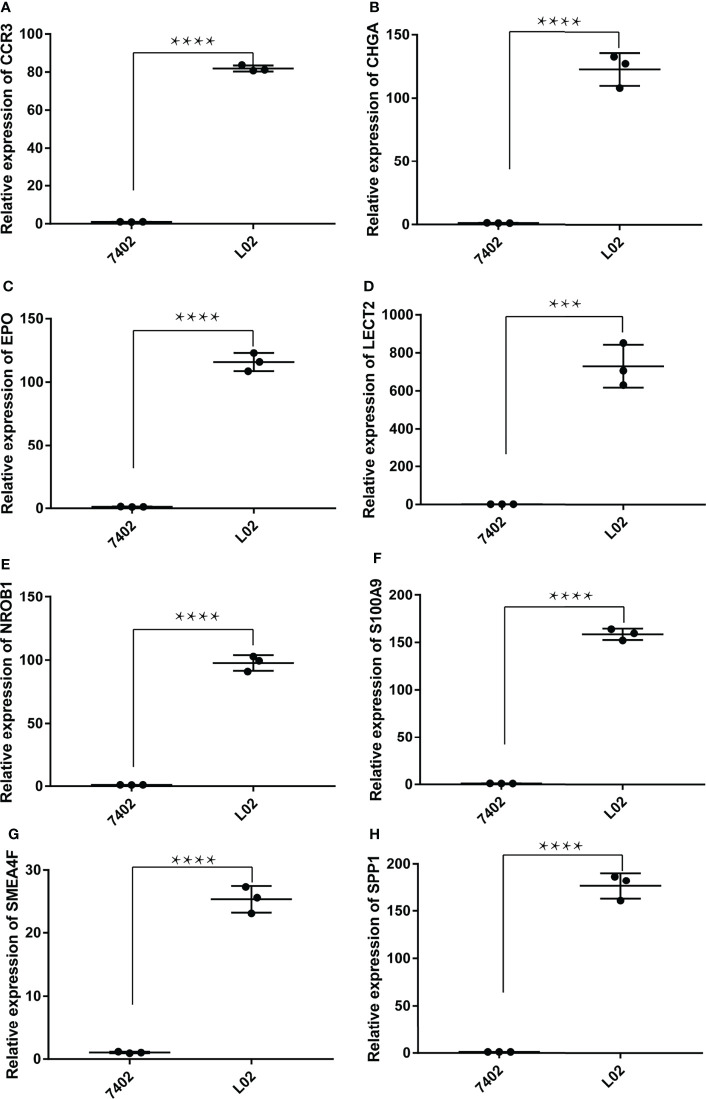
The relative RNA expression level of LECT2, SEMA4F, EPO, CHGA, NR0B1, S100A9, CCR3, and SPP1. The RNA expression of CCR3** (A)**, CHGA** (B)**, EPO **(C)**, LECT2** (D)**, NR0B1** (E)**, S100A9** (F)**, SEMA4F** (G)** and SPP1** (H)** were low expressed in human hepatoma cells compared with normal human hepatic epithelial cells (Unpaired t-test, ***P < 0.001, ****P < 0.0001).

The RNA expression of LECT2, SEMA4F, EPO, CHGA, NR0B1, S100A9, CCR3, and SPP1 in normal human hepatic epithelial cells HL-02 and human hepatoma cells BEL_7402 were compared by qPCR. It was found that LECT2, SEMA4F, EPO, CHGA, NR0B1, S100A9, CCR3, and SPP1 were low expressed in human hepatoma cells compared with normal human hepatic epithelial cells (Unpaired t-test, *p*<0.01) (**Figure 8**).

## Discussion

The current clinical problems of liver cancer are mainly associated with untimely diagnosis and treatment, which can be attributed to a special double blood supply structure of the liver allowing the formation of a microenvironment providing autoimmune tolerance (50). This phenomenon, together with the immune escape of liver cancer cells, indicates that targeted immune-therapeutics should be an effective treatment for HCC (51). In recent years, the research on the mechanism of liver cancer immunotherapy has made great progress (52), but there are still many challenges. Our study identified an immunity-related eight-gene signature that can be used as an independent prognostic indicator of the LIHC outcome, offering a quantitative clinical method to predict patient’s survival. Analysis of the association between LIHC prognosis and immune cell infiltration showed that each gene in the immune-related eight-gene signature was strongly related to M0 macrophage infiltration.

Our analysis of TCGA database indicated that the mutation frequency of the TP53 gene was the highest among the genes mutated in LIHC, and GSEA and GSVA revealed important pathways enriched in patients harbouring TP53 mutations. We also determined an immunity-related eight-gene prognostic signature and performed analysis of its association with immune cell infiltration, which showed that the infiltration of M1 macrophages, resting CD4+ memory T cells, activated NK cells, and CD8+ T cell suggested a better prognosis. Previous reports indicate that liver cancer tissues are characterized with a high expression level of PD-L1, CTLA4, lymphocyte activation gene 3, and other immunosuppressive molecules, which is negatively associated with the tumour infiltration of IFNγ+ T lymphocytes. Antibody treatment could increase the rate of CD8+ tumour-infiltrating T lymphocytes and the production of cytokines in liver cancer tissues (53). The infiltration of T cells before and after immunotherapy could be used to evaluate the effect of drugs enhancing the response to immune checkpoint blockers and to determine whether T cell infiltration by itself could predict the outcome of immunotherapy (54). It was reported that ependymin related protein 1 (EPDR1) and BRCA1 are correlated with immune cell infiltration and prognosis in HCC (55, 56). Recently, a nine immune-related gene model with an independent prognostic capability for HCC has been developed and shown to be associated with immune cell infiltration (57).

Using univariate Cox regression, we analysed the relationship among immune-associated genes and the prognosis of patients with LIHC, which were then divided into groups according to risk scores, and the optimal prognostic signature containing eight genes was established using iterative Lasso Cox regression analysis. ROC analysis performed in the external validation set revealed that the immune-associated eight-gene signature had a significant ability to predict 3-, 5-, and 7-year prognosis for patients with LIHC. Furthermore, compared with common prognostic biomarkers of LIHC, the eight-gene signature showed a superior predictive power and was proved to be an independent prognostic predictor of patient survival. We constructed a nomogram combining the clinicopathological characteristics and the immune-related eight-gene signature to offer clinicians a quantitative method for predicting the LICH outcome, which should aid in the selection of optimal treatment approaches. Analysis of immune cell infiltration revealed that Tregs, activated NK cells, and M0 macrophages had the highest, whereas activated dendritic cells – the lowest infiltration rate in the high-risk group.

The immunity-related genes composing our eight gene prognostic signature have been previously shown to be involved in oncogenesis. Thus, SEMA4F encoding semaphorin 4F plays a role in axonal growth cone guidance (58) and induction of neurogenesis in prostate cancer (59). The expression of the erythropoietin-encoding EPO gene is related to apoptosis, survival, and proliferation in the early stages of clear cell renal cell carcinoma (60) and has been identified as a distinct prognostic factor for overall and metastases-free survival and locoregional control in locally advanced HNSCC (61). The overexpression of S100A9 encoding calgranulin B has been suggested to play a vital role in the progress of oral squamous cell carcinoma (OSCC) and may serve as a diagnostic and prognostic biomarker for OSCC (62) and nonsmall-cell lung carcinoma (63).

In order to determine the association between LIHC prognosis and immune cell infiltration, we constructed a correlation heat map, which showed that seven genes in the immune-related eight-gene signature were negatively associated with the infiltration of M0 macrophages, resting mast cells, and Tregs. Previous studies have shown that S100A9 plays a significant role in the regulation of immune response and inflammation in most tumours and that it promotes cancer metastasis by accelerating tumour cell proliferation and invasion (64–66), which is consistent with the role of tumour-associated inflammation in supporting metastasis and cancer progression (66–68). Leukocyte cell-derived chemotaxin-2, a 16-kDa secreted protein encoded by the LECT2 gene (69), is involved in the regulation of the tumour microenvironment (70) and plays a critical role in hepatic oncogenesis. Thus, LECT2 deletion modifies the tumour microenvironment and alters cancer phenotypes, suggesting that it is a promising immunotherapeutic target in liver cancer (71). Another study has found that LECT2 expression in HCC is strongly correlated with tumour angiogenesis (72).

It was shown that the expression of C motif chemokine receptor 3 (CCR3) was correlated with malignancy of tumour cells (73). CCR3 ligand may be up-regulated by tumour-related inflammation and involved in the progress of renal cell carcinoma (74), whereas the CCR3/eotaxin-1 loop could induce malignant cell growth in T-cell lymphomas (75, 76). Secreted phosphoprotein 1 (SPP1), also known as osteopontin, is a multifunctional protein first characterized as a biomarker in epithelial cell transformation (77) and suggested to function as an enhancer of HCC growth targeted by miR-181c, thus representing a potential candidate biomarker for HCC diagnosis and therapy (78). We also found that LECT2, SEMA4F, EPO, CHGA, NR0B1, S100A9, CCR3, and SPP1 were low expressed in hepatocellular carcinoma, which may be new cancer therapeutic targets.

## Data Availability Statement

The datasets presented in this study can be found in online repositories. The names of the repository/repositories and accession number(s) can be found in the article/**Supplementary Material**.

## Author Contributions

RC analyzed the data and wrote the manuscript. MZ designed the framework of the paper. YA, DL, and QT interpreted data. GT revised the manuscript. The final version of the manuscript has been approved by all authors.

## Funding

We are grateful for the support of National Natural Science Foundation of China (81827805), National Key R&D Program of China (2018YFA0704100, 2018YFA0704100), the Jiangsu Provincial Medical Youth Talent (QNRC2016816), the Project of Jiangsu Provincial Health and Family Planning Commission (H2018090).

## Conflict of Interest

The authors declare that the research was conducted in the absence of any commercial or financial relationships that could be construed as a potential conflict of interest.

## Publisher’s Note

All claims expressed in this article are solely those of the authors and do not necessarily represent those of their affiliated organizations, or those of the publisher, the editors and the reviewers. Any product that may be evaluated in this article, or claim that may be made by its manufacturer, is not guaranteed or endorsed by the publisher.

## Supplementary Material

Supplementary Figure 1 Comparison of the TP53 and NO_TP53 groups using GSEA and GSVA. **(A)** File ‘c2.all.v7.1.symbols.gmt’ was used as the reference gene set for GSEA. **(B)** File ‘h.all.v7.1.symbols.gmt’ was used as the reference gene set for GSVA.

Supplementary Figure 2 Single-factor Cox regression model.

Supplementary Figure 3 Confirmation of the optimal gene signature using an external validation set. **(A)** A centralized risk factor correlation diagram for the validation set. **(B)** Kaplan-Meier analysis of the correlation between the eight-gene signature and patient survival. **(C)** ROC analysis of 3-, 5-, and 7-year prognosis for patients with LIHC. **(D)** Time-conditioned ROC curve of the eight-gene signature and common prognostic biomarkers of LIHC.

Supplementary Figure 4 Immune cell infiltration analysis. **(A)** Correlations in immune cell infiltration. **(B)** Immune cell interaction network; circle size (large to small) is proportional to the intensity of interaction (strong to weak). **(C)** Differences in immune cell infiltration between high- and low-risk groups. **(D)** Immune cell composition analysis.
