# Supplementary figures and images for: A Prognostic Gene Signature for Hepatocellular Carcinoma
Source: Front Oncol. 2022 Apr 27;12:841530. doi: 10.3389/fonc.2022.841530 (PMC9091376; doi:10.3389/fonc.2022.841530)

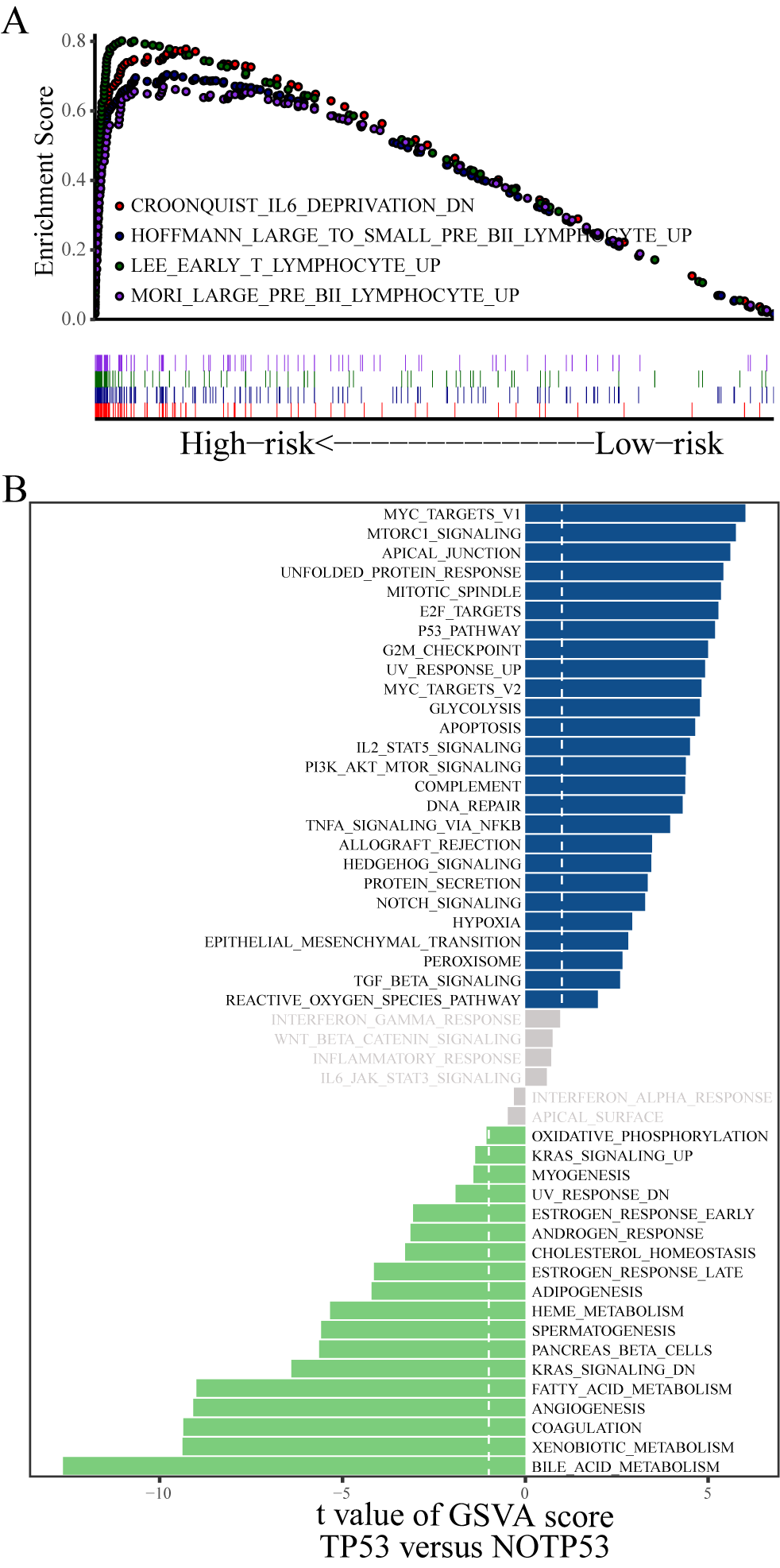

Supplement: Supplementary Figure 1 — Comparison of the TP53 and NO_TP53 groups using GSEA and GSVA. (A) File ‘c2.all.v7.1.symbols.gmt’ was used as the reference gene set for GSEA. (B) File ‘h.all.v7.1.symbols.gmt’ was used as the reference gene set for GSVA. [file Image_1.tif]

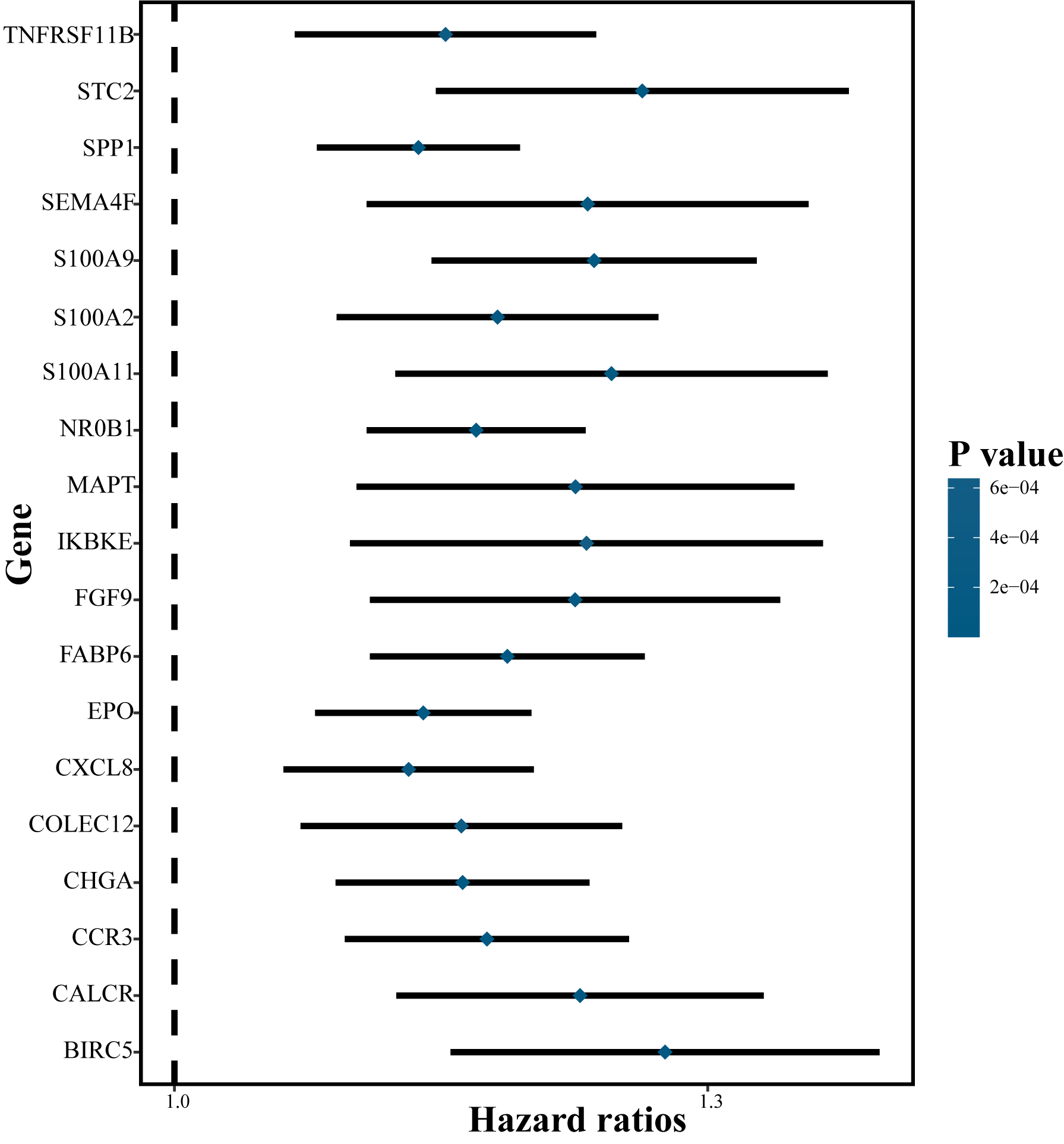

Supplement: Supplementary Figure 2 — Single-factor Cox regression model. [file Image_2.tif]

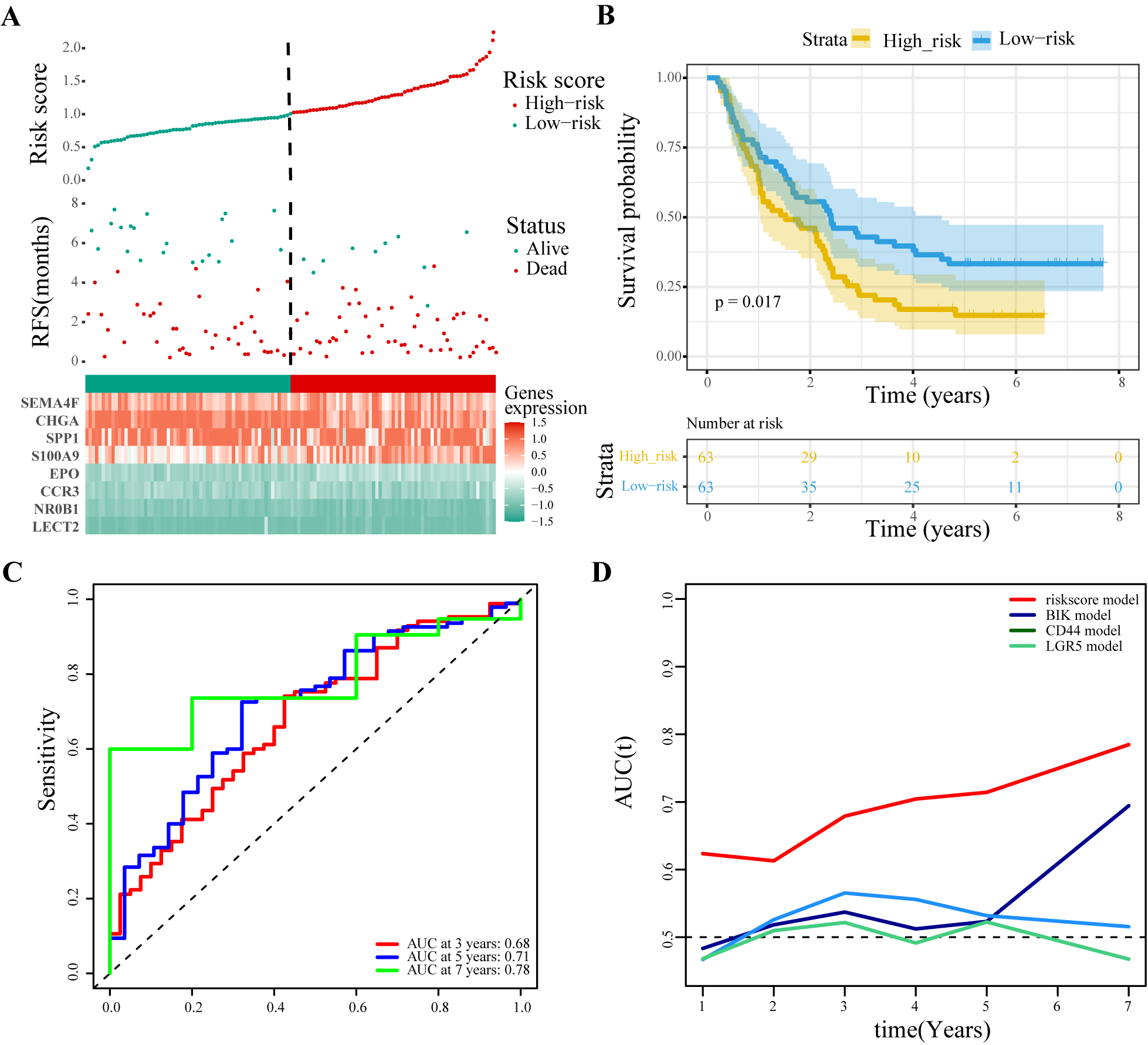

Supplement: Supplementary Figure 3 — Confirmation of the optimal gene signature using an external validation set. (A) A centralized risk factor correlation diagram for the validation set. (B) Kaplan-Meier analysis of the correlation between the eight-gene signature and patient survival. (C) ROC analysis of 3-, 5-, and 7-year prognosis for patients with LIHC. (D) Time-conditioned ROC curve of the eight-gene signature and common prognostic biomarkers of LIHC. [file Image_3.tif]

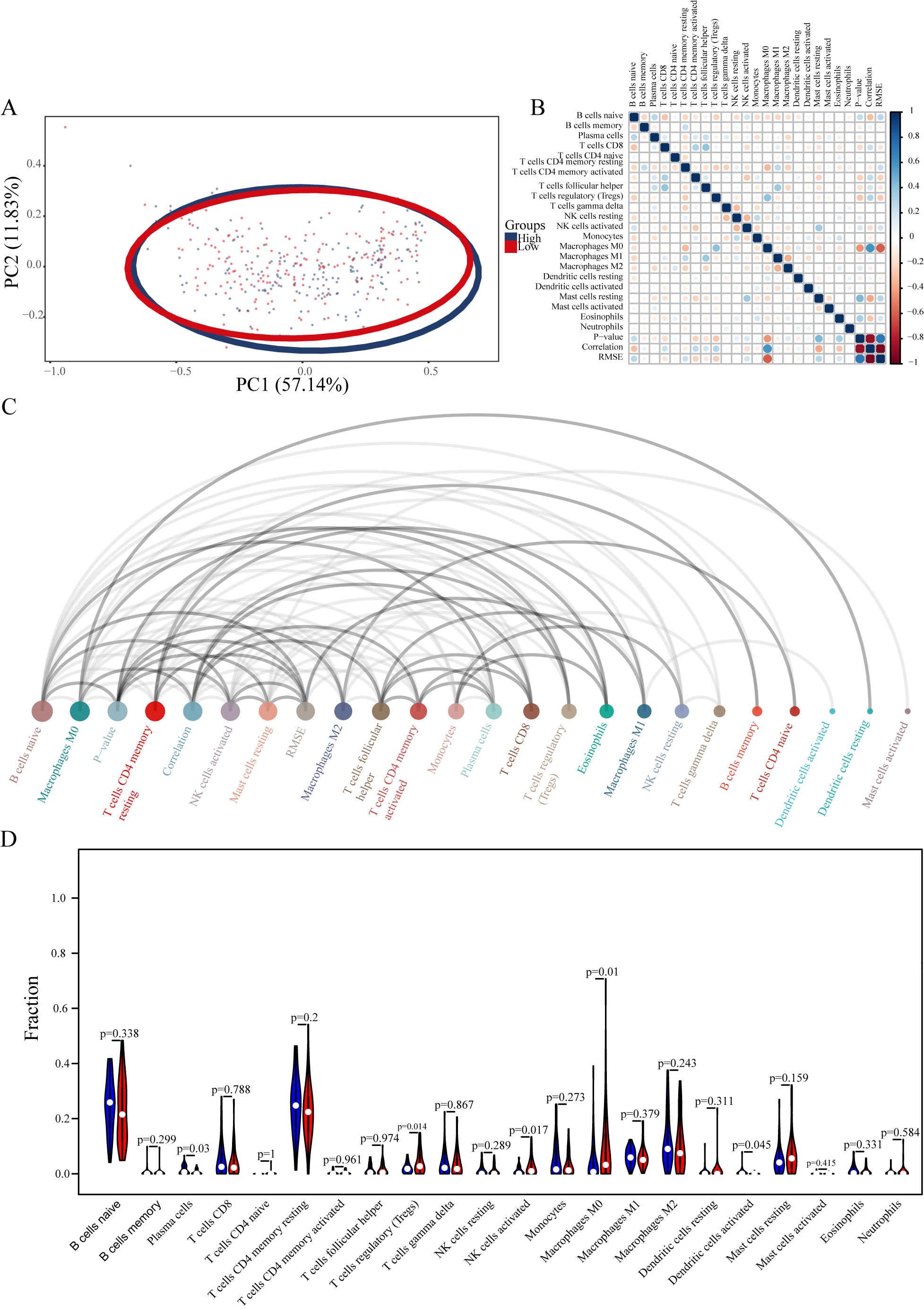

Supplement: Supplementary Figure 4 — Immune cell infiltration analysis. (A) Correlations in immune cell infiltration. (B) Immune cell interaction network; circle size (large to small) is proportional to the intensity of interaction (strong to weak). (C) Differences in immune cell infiltration between high- and low-risk groups. (D) Immune cell composition analysis. [file Image_4.tif]
